# Longitudinal Associations Between Friendship Support, Strain, and Loneliness Among Older Adult Couples

**DOI:** 10.1093/geroni/igaf122.1940

**Published:** 2025-12-31

**Authors:** Ashley Ermer, Jeremy Kanter

**Affiliations:** Montclair State University, Montclair, New Jersey, United States; University of Illinois Urbana-Champaign, Urbana, Illinois, United States

## Abstract

Friendship plays an important role in later life. How friendship operates in the context of marriage, along with its links to loneliness, is underexplored. The life course perspective, which highlights time and linked lives, serves as a theoretical framework for this study. Specifically, the role of historical time (i.e., Great Recession), chronological time, and linked lives (spouses’ friendship support, friendship strain, and loneliness) will be considered. The purpose of this study is to examine the longitudinal associations between spouses’ friendship support, friendship strain, and loneliness over 12 years. Waves 8 (2006), 10 (2010), 12 (2014), and 14 (2018) of the Leave-Behind Questionnaire in the Health and Retirement Study were utilized (N = 849). Using two dyadic, random-intercept cross-lagged panel model (RI-CLPM; separate models for friendship strain and friendship support), out of a total of 96 associations, 21 actor and partner effects were statistically significant across both models. For example, women’s greater loneliness in 2010 was associated with men’s lower friendship support in 2014. Men’s greater friendship strain in 2010 was associated with women’s greater loneliness in 2014. Further analyses will probe whether significant effects are the same over time and/or differ between spouses. Implications regarding the role that individuals’ social networks play in their spouse’s loneliness will be discussed, along with future directions for research.

